# Predictive Nomogram for Recurrence After Upfront Surgery for Resectable Pancreatic Ductal Adenocarcinoma: A Multicenter Study (OS-HBP-2)

**DOI:** 10.3390/cancers18071181

**Published:** 2026-04-07

**Authors:** Ryuichi Yoshida, Kosei Takagi, Kazuya Yasui, Masayoshi Hioki, Takehiro Okabayashi, Toru Kojima, Yoshikatsu Endo, Daisuke Nobuoka, Kenta Sui, Masaru Inagaki, Susumu Shinoura, Masashi Kimura, Tatsuo Matsuda, Hideki Aoki, Toshiyoshi Fujiwara

**Affiliations:** 1 Department of Gastroenterological Surgery, Okayama University Graduate School of Medicine, Dentistry, and Pharmaceutical Sciences, Okayama 700-8558, Japan; ryuichi-yoshida@cc.okayama-u.ac.jp (R.Y.); pjyv6nvp@s.okayama-u.ac.jp (K.Y.); toshi_f@md.okayama-u.ac.jp (T.F.); 2 Department of Surgery, Fukuyama City Hospital, Hiroshima 721-8511, Japan; kahioki@city.fukuyama.hiroshima.jp (M.H.); nbokdisk5@gmail.com (D.N.); 3 Department of Surgery, Kochi Health Sciences Center, Kochi 781-0111, Japan; takehiro_okabayashi@khsc.or.jp; 4 Department of Surgery, Okayama Saiseikai General Hospital, Okayama 700-8511, Japan; trkojima@okayamasaiseikai.or.jp; 5 Department of Surgery, Himeji Red Cross Hospital, Hyogo 670-8540, Japan; y-endou@himeji.jrc.or.jp; 6 Department of Surgery, Kagawa Prefectural Hospital, Kagawa 760-8557, Japan; suikenta25@ybb.ne.jp; 7 Department of Surgery, National Hospital Organization Fukuyama Medical Center, Hiroshima 720-0825, Japan; inagaki.masaru.dp@mail.hosp.go.jp; 8 Department of Surgery, Tsuyama Chuo Hospital, Okayama 708-0841, Japan; s.shino@tch.or.jp; 9 Department of Surgery, Matsuyama Shimin Hospital, Ehime 790-0067, Japan; kishimrms6717@matsuyama-shimin-hsp.or.jp; 10 Department of Surgery, Tenwakai Matsuda Hospital, Okayama 710-0056, Japan; tmatsuda1004@gmail.com; 11 Department of Surgery, National Hospital Organization Iwakuni Clinical Center, Yamaguchi 740-0037, Japan; aoki.hideki.hy@mail.hosp.go.jp

**Keywords:** pancreatic cancer, resectable, upfront surgery, recurrence, nomogram

## Abstract

**Background/Objectives**: Postoperative recurrence is a critical issue in the treatment of resectable pancreatic ductal adenocarcinoma (rPDAC). Moreover, the prognosis after early recurrence is extremely poor. This study aimed to develop a recurrence prediction model and to define early recurrence after upfront surgery (UFS) for rPDAC. **Methods**: This multicenter retrospective study included patients who underwent UFS for anatomically rPDAC between January 2013 and December 2017. Multivariate analyses were conducted to identify the risk factors for recurrence-free survival and to construct a recurrence prediction model. Subsequently, a minimum *p* value approach was used to determine the optimal cutoff values for early and late recurrence. **Results**: The cohort included 603 patients (325 men and 278 women). During the median follow-up period of 25 months (interquartile range, 15–38 months), 381 patients (63.2%) experienced a recurrence. Multivariate analyses revealed carbohydrate antigen 19-9 ≥37 U/mL (hazard ratio [HR], 1.58; *p* < 0.001), tumor size ≥ 2.2 cm (HR, 1.59; *p* < 0.001), lymph node metastasis (HR, 1.86; *p* < 0.001), R1 resection (HR, 1.56; *p* = 0.002), and no adjuvant chemotherapy (HR, 1.54; *p* < 0.001) as independent predictors. The recurrence prediction model demonstrated an area under the curve of 0.72–0.75. The optimal threshold for early and late recurrences was a recurrence-free interval of five months. Carbohydrate antigen 19-9 ≥ 156 U/mL was a significant predictor of early recurrence (OR, 3.28; *p* < 0.001). **Conclusions**: This study identified the prognostic risk factors for recurrence and developed a recurrence prediction model for patients undergoing UFS for rPDAC. Moreover, a recurrence-free interval of five months was identified as the optimal threshold for distinguishing between early and late recurrences.

## Simple Summary

Despite recent advances in multidisciplinary treatment, postoperative recurrence remains a major issue for patients with resectable pancreatic ductal adenocarcinoma (rPDAC). This multicenter study (*n* = 603) investigated prognostic factors for recurrence in patients undergoing upfront surgery for rPDAC. We developed a recurrence prediction model using tumor markers, tumor size, lymph node metastasis, margin status, and adjuvant chemotherapy. Moreover, a recurrence-free interval of five months was identified as the optimal threshold for early and late recurrences. Preoperative risk assessment is essential for evaluating recurrence risk and prognosis in patients with rPDAC.

## 1. Introduction

Pancreatic ductal adenocarcinoma (PDAC) is a highly aggressive and lethal malignancy with a poor survival rate [1]. Although surgery is the only potentially curative treatment for PDAC, recurrence after surgery remains a major issue in patients with PDAC, with approximately 40–50% experiencing early recurrence within 12 months after surgery [2]. As upfront surgery (UFS) is recommended for patients with resectable PDAC (rPDAC) [3], investigation of the risk factors for recurrence is required. Moreover, early recurrence has been variably defined as recurrence occurring within 6–12 months of surgery [2,4]. Since biological behavior seems to differ between early and late recurrences after surgery [2,4], further attempts should be made to define and predict early recurrence in PDAC.

PDAC resectability is currently classified into three categories: resectable, borderline resectable, and unresectable [3]. Therefore, resectability-specific analyses are needed to predict early recurrence. To date, several nomograms have been developed to predict early recurrence of rPDAC [5,6]. Our group previously proposed a prognostic prediction model for survival after UFS in patients with rPDAC using a multicenter database from the Okayama Study Group of HBP Surgery (OS-HBP-2) [7]. However, a clinically useful prediction model for recurrence and a clear definition of early recurrence of rPDAC remain unresolved.

This post hoc analysis of OS-HBP-2 aimed to develop a prediction model for recurrence after UFS in patients with rPDAC. In addition, early recurrence was defined based on the post-recurrence survival (PRS) after UFS.

## 2. Materials and Methods

### 2.1. Cohort

This multicenter, retrospective study included patients who underwent UFS for anatomically rPDAC at any location between January 2013 and December 2017. Clinical data were collected from 15 hospitals in the OS-HBP (Okayama University Hospital, Fukuyama City Hospital, Kochi Health Sciences Center, Hiroshima City Hiroshima Citizens Hospital, Okayama Saiseikai General Hospital, Himeji Red Cross Hospital, Kagawa Prefectural Hospital, National Hospital Organization Fukuyama Medical Center, Tsuyama Chuo Hospital, Matsuyama Shimin Hospital, Tenwakai Matsuda Hospital, National Hospital Organization Iwakuni Medical Center, Himeji St. Maria Hospital, Japanese Red Cross Kobe Hospital, and Okayama Rosai Hospital). Patients with borderline anatomically rPDAC, Lewis negativity, and postoperative in-hospital mortality were excluded. The study was approved by the Ethics Committee of our institution (approval no. 2211-039) and conducted in accordance with the principles of the Declaration of Helsinki. The requirement for informed consent was waived due to the retrospective nature of this study.

### 2.2. Clinical Data

The following data were collected using the database of the OS-HBP-2: gender; age; body mass index; preoperative biliary drainage; carbohydrate antigen 19-9 (CA 19-9); the modified Glasgow Prognostic Score calculated by C-reactive protein and serum albumin levels (zero, one, or two) [8]; preoperative radiological findings (tumor location, tumor size, venous involvement [no tumor contact with the portal vein [PV] or superior mesenteric vein [SMV] or tumor contact with the PV or SMV of ≤180° [9], and lymph node metastasis); type of surgery (pancreaticoduodenectomy, distal pancreatectomy, or total pancreatectomy); PV or SMV resection; operative time; estimated blood loss; pathological findings, including R status [10], evaluated by the Union for International Cancer Control seventh edition [11]; postoperative outcomes (major complications defined by the Clavien–Dindo classification ≥ grade 3 [12] and postoperative pancreatic fistula [≥grade B] [13]); adjuvant chemotherapy; and long-term outcomes (recurrence and status at last follow-up [survival or death]).

The initial anatomical resectability status was evaluated based on radiological findings at a multidisciplinary conference using the National Comprehensive Cancer Network criteria for rPDAC: no tumor contact with major arterial structures (celiac artery, superior mesenteric artery, and/or common hepatic artery) and no tumor contact with the PV or SMV, with ≤180° contact without vein contour irregularity [3]. The standard protocol for adjuvant chemotherapy includes S-1 or gemcitabine for six months after surgery [14]. Recurrence patterns included liver, peritoneal, local, lung, and multiple metastases.

### 2.3. Literature Search

A comprehensive literature search of PubMed was performed on 13 January 2026. The search terms used were pancreatic cancer, upfront surgery, recurrence, and nomogram. Relevant articles were identified through a manual review of the reference lists. The search was limited to articles published in English, without any limitations regarding the year of publication.

### 2.4. Statistical Analysis

Continuous variables are presented as medians with interquartile ranges (IQRs), and categorical variables are presented as proportions. Differences between groups were assessed using the Mann–Whitney U test for continuous variables and either Fisher’s exact test or the chi-squared (χ^2^) test for categorical variables. All statistical analyses were performed using the JMP (version 11; SAS Institute, Cary, NC, USA) and EZR software (version 1.65; Saitama Medical Center, Jichi Medical University, Saitama, Japan).

Overall survival (OS), recurrence-free survival (RFS), and PRS rates were estimated using the Kaplan–Meier method, and differences between survival curves were analyzed using the log-rank test. OS, RFS, and PRS were defined as the time intervals from surgery to death or last follow-up, from surgery to recurrence, death, or last follow-up, and from recurrence to death or last follow-up, respectively. Univariate and multivariate Cox regression analyses were performed to identify prognostic factors associated with RFS in all cohorts, and hazard ratios (HRs) and 95% confidence intervals (CIs) were reported. Based on the results of the multivariate analyses, a prognostic prediction model for RFS was developed. The performance of the prediction model was assessed by calculating the area under the receiver operating characteristic curve (AUC). Subsequently, the bootstrap method was employed to perform internal validation and evaluate the discriminative performance [15]. Calibration curves and concordance indices were calculated to assess the predictive validity of the model. Moreover, a decision curve analysis was performed to evaluate the prediction model for RFS.

Subsequently, a minimum *p* value approach using the log-rank test was adopted to determine the optimal cutoff value for early and late recurrences based on the length of the PRS [4]. Using this approach, the lowest *p* value was selected to evaluate the cutoff values. In the subgroup analysis, preoperative predictors associated with early recurrence were investigated in the recurrence cohort using multivariate logistic regression models. Odds ratios (ORs) and 95% CIs were calculated.

## 3. Results

### 3.1. Study Cohort

Among the 647 patients, 44 individuals, comprising those with Lewis negativity (*n* = 41) and postoperative in-hospital mortality (*n* = 3), were excluded. The clinicopathological characteristics of 603 patients are presented in Table 1. The cohort included 325 men and 278 women, with a median age of 72 years (IQR, 65–77 years) and a CA 19-9 level of 111 U/mL (IQR, 30–392 U/mL). Preoperative radiological findings revealed tumor contact with the PV/SMV of ≤180° in 174 patients (28.9%). The pathological findings revealed the following T and N stages: T1 (*n* = 48, 8.0%), T2 (*n* = 31, 5.1%), T3 (*n* = 524, 86.9%), and lymph node metastasis (*n* = 331, 54.9%).

During the median follow-up of the entire cohort of 25 months (IQR, 15–38 months), 381 (63.2%) patients experienced recurrence, whereas 222 (36.8%) did not. The recurrence patterns were liver (*n* = 113), peritoneum (*n* = 43), local (*n* = 88), lung (*n* = 45), and multiple (*n* = 83) lesions. The median OS and RFS were 34 months (95% CI, 32–42 months) and 15 months (95% CI, 13–17 months), respectively.

Baseline characteristics stratified by recurrence status are described in Table 1. The recurrence group had significantly higher CA 19-9 levels, larger tumor size, and higher incidences of tumor contact with the PV/SMV of ≤180°. Although the preoperative clinical stage did not differ significantly between the groups, the pathological stage was significantly more advanced in the recurrence group.

### 3.2. Prognostic Factors Associated with RFS

The results of the univariate and multivariate analyses investigating the predictors for RFS after UFS in all cohorts (*n* = 603) are shown in Table 2. Multivariate analyses identified CA 19-9 ≥ 37 U/mL (HR, 1.58; *p* < 0.001), tumor size ≥ 2.2 cm (HR, 1.59; *p* < 0.001), lymph node metastasis (HR, 1.86; *p* < 0.001), R1 resection (HR, 1.56; *p* = 0.002), and no adjuvant chemotherapy (HR, 1.54; *p* < 0.001) as independent prognostic factors associated with RFS.

### 3.3. Recurrence Prediction Model Following UFS

The predictive probability of RFS was constructed using the results of multivariate analyses (Table 2 and Equation (1); available in Supplementary Digital Content S1).

S(t∣x) = [S0(t)]^exp(linear predictor)

= [exp(−0.005356 × t)]^exp(0.484579 × [tumor size ≥ 2.2 cm] + 0.684197 × [Lymph node metastases] + 0.457116 × [CA 19-9 ≥ 37] + 0.460316 × [R1] + 0.489966 × [No adjuvant chemotherapy])

(1) = exp(−0.005356 × t × exp{0.484579 × [tumor size ≥ 2.2 cm] + 0.684197 × [Lymph node metastases] + 0.457116 × [CA 19-9 ≥ 37] + 0.460316 × [R1] + 0.489966 × [No adjuvant chemotherapy])

p (predictive probability; %) = S(t∣x) × 100

S(t∣x) survival function at time t for an individual with covariates x

S0(t) baseline survival function

### 3.4. Model Performance and Calibration of the Model

**Figure 1:**
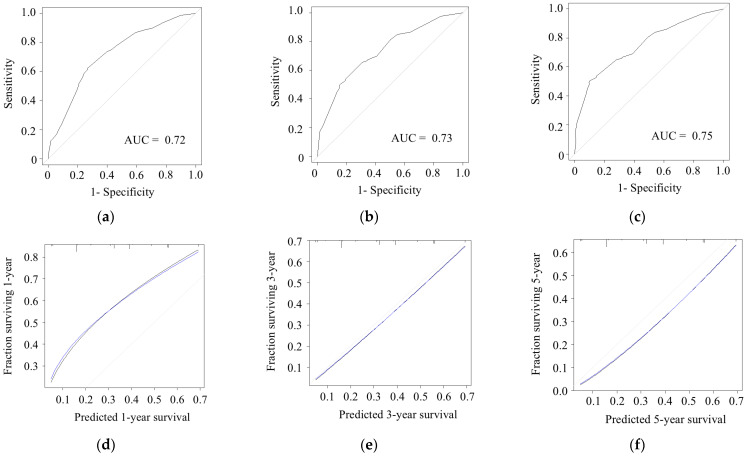
Model performance and calibration of the model. Receiver operating characteristic curves and area under the curve for the model predicting (**a**) 1-year, (**b**) 3-year, and (**c**) 5-year recurrence-free survival. Calibration plots of the model for (**d**) 1-year, (**e**) 3-year, and (**f**) 5-year recurrence-free survival. Black line, observed; gray line, ideal; blue line, optimism corrected. AUC, area under the curve.

The receiver operating characteristic curve of the prediction model is shown in Figure 1. The AUC values for 1-, 3-, and 5-year RFS were 0.72 (Figure 1a), 0.73 (Figure 1b), and 0.75 (Figure 1c), respectively, indicating good discriminative ability in this clinical setting. The calibration plots generated using the bootstrap method are also shown in Figure 1. The Brier scores were 0.138 (Figure 1d), 0.178 (Figure 1e), and 0.177 (Figure 1f) at 1, 3, and 5 years, respectively, suggesting stable predictive performance of the model over time despite a slight increase in accuracy at longer follow-up.

### 3.5. Decision Curve Analysis of the Prediction Model

**Figure 2:**
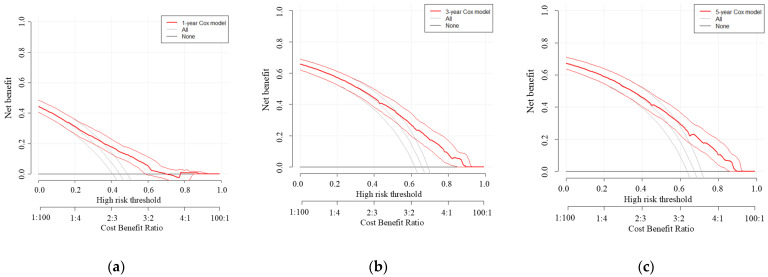
Decision curve analysis of the prediction model for (**a**) 1-, (**b**) 3-, and (**c**) 5-year RFS. The red line represents the net benefit of the prediction model, while the gray and black lines represent the treat-all and treat-none strategies, respectively. The *x*-axis shows the threshold probability, which reflects the risk level at which a clinician would opt for intervention, and the corresponding cost–benefit ratios are displayed along the secondary axis. The *y*-axis represents net benefit, incorporating the trade-off between true positives and false positives.

Figure 2 shows the results of a decision curve analysis of the prediction model for 1-, 3-, and 5-year RFS. The model provided a higher net benefit than both alternative strategies across a range of threshold probabilities at 1, 3, and 5 years, with a higher-risk-threshold range of clinical usefulness observed at 3 and 5 years.

### 3.6. Definition of Early and Late Recurrence

**Figure 3:**
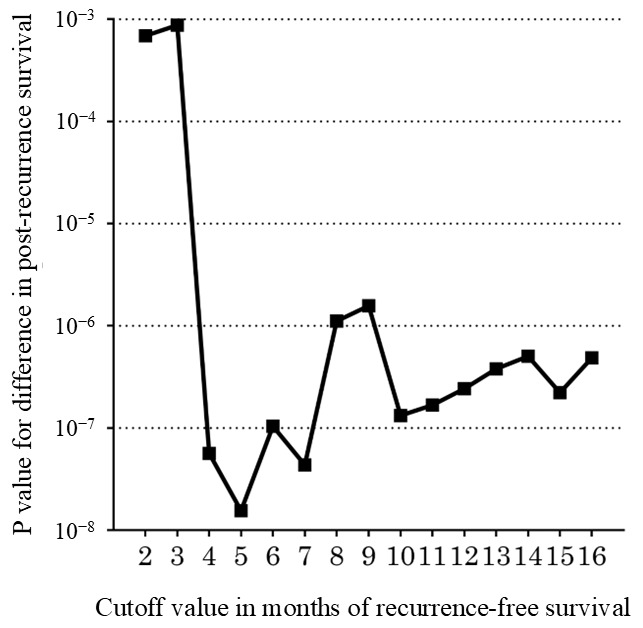
Association between different cutoff values of recurrence-free survival and corresponding *p* values in post-recurrence survival (PRS). The optimal cutoff value for early and late recurrence based on the length of PRS was 5 months.

The results of evaluating the cutoff values for determining early and late recurrence based on PRS are shown in Table 3. The optimal cutoff value for early and late recurrence based on the PRS was 5 months (Figure 3).

### 3.7. Outcomes Stratified by Early and Late Recurrence

**Figure 4:**
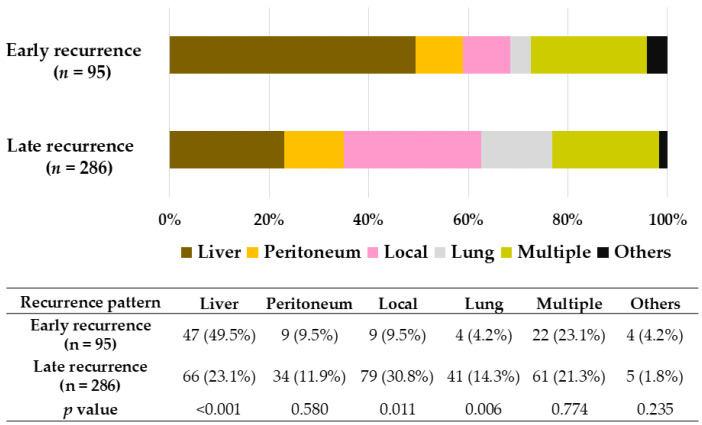
Recurrence patterns in the early and late recurrence groups.

Using a cutoff value of 5 months, patients with recurrence were divided into early (*n* = 95) and late (*n* = 286) recurrence groups. The recurrence patterns in the early and late recurrence groups are shown in Figure 4. Recurrence patterns differed significantly between the groups, with a higher incidence of liver metastases in the early recurrence group (*p* < 0.001) and local recurrences in the late recurrence group (*p* < 0.001).

The clinicopathological characteristics of the recurrence cohort are presented in Table 4. The early recurrence group had significantly higher CA 19-9 levels, larger tumor sizes, and less frequent administration of adjuvant chemotherapy.

**Figure 5:**
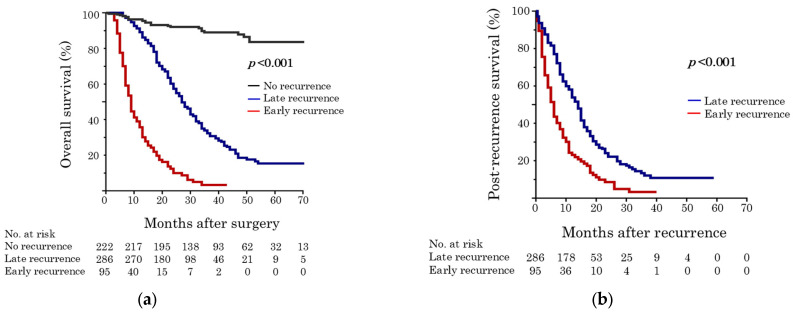
(**a**) Overall survival curves among patients with no, early, and late recurrence in all cohorts; (**b**) post-recurrence survival in the recurrence cohort.

The OS rates of patients with no, early, and late recurrence are shown in Figure 5a. The 3-year OS rates were 89.0%, 32.4%, and 3.3% in patients with no, late, and early recurrence, respectively (*p* < 0.001). The early recurrence group had significantly worse median PRS than the late recurrence group (6 vs. 14 months, *p* < 0.001; Figure 5b).

### 3.8. Prognostic Factors Associated with Early Recurrence

In a subgroup analysis of the recurrence cohort (*n* = 381), multivariate analyses identified CA 19-9 ≥156 U/mL as a significant predictor of early recurrence (OR, 3.28; *p* < 0.001; Table 5).

### 3.9. Literature Review

A literature search identified eight relevant studies that reported nomograms for predicting recurrence after surgery for PDAC (Table 6) [5,6,16,17,18,19,20]. Four single-center and four multicenter retrospective studies were included. Several perioperative parameters were used in the nomograms, with reported AUC values ranging from 0.655 to 0.941.

## 4. Discussion

Current guidelines recommend UFS for patients with rPDAC [3]; however, the high incidence of recurrence adversely affects prognosis after surgery. This multicenter study investigated the risk factors for recurrence after UFS and developed a prediction model for recurrence after UFS in patients with rPDAC. Using a risk prediction model for RFS including five parameters (CA 19-9 level, tumor size, lymph node metastasis, margin status, and adjuvant chemotherapy), the risk of RFS after UFS for rPDAC can be estimated. Moreover, we found that five months was the most appropriate threshold for defining early recurrence after UFS for rPDAC.

The recurrence rate in this study was comparable to previously reported findings, with a postoperative recurrence rate of 50–60% [6]. Preoperative tumor markers and tumor size are well-known risk factors for postoperative PDAC recurrence [2]. Moreover, pathological and postoperative factors, including lymph node metastasis, margin status, and adjuvant chemotherapy, have been reported to be important predictors for prognosis and recurrence [21,22,23,24]. Using the prognostic risk factors for RFS identified in the multivariate analyses (Table 2), a simple recurrence prediction model was constructed. The predictive probability of RFS was calculated using this model. The model performance, with an AUC of 0.72–0.75, showed good discriminative ability, and the discriminative performance of the model was considered acceptable.

As summarized in Table 6, previous studies have developed nomograms to predict early recurrence using various predictive parameters, including tumor markers, tumor characteristics, and radiological findings. However, different cutoff values for early recurrence have been used, such as 6 and 12 months [5,6,16,17,18,19,20]. Although a landmark study reported that 12 months was the optimal threshold for differentiating between early and late recurrence in patients with resected PDAC [4], the risk of recurrence differed between patients with resectable and borderline resectable PDAC. Therefore, this study employed a minimum *p* value approach using the log-rank test to determine the optimal cutoff values for early and late recurrence after UFS, focusing on rPDAC. We found a recurrence-free interval of five months to be the optimal cutoff value for early and late recurrence of rPDAC (Figure 3).

Regarding recurrence patterns, the early recurrence group had a higher incidence of systemic recurrences such as liver metastases. In contrast, the late recurrence group experienced locoregional recurrence more frequently (Figure 4), which is consistent with previously reported findings [5,18]. PDAC is a systemic disease characterized by micrometastases in its early stages [25]. As CA 19-9 levels reflect biological and conditional dimensions [26,27], higher CA 19-9 levels may be associated with early recurrence in the recurrence cohort (Table 4). Moreover, OS was significantly stratified among patients with no, late, or early recurrences, showing a worse prognosis in the early recurrence group (Figure 5a). Although previous studies have compared outcomes between the early and non-early recurrence groups [5,6,17,18,20], the non-early recurrence group included patients with no recurrence and those with late recurrence. Therefore, this study first investigated the risk factors for recurrence after UFS in the entire cohort (Table 2) and then investigated the predictors of early recurrence in the recurrence cohort (Table 5). Interestingly, only an elevated CA 19-9 level (≥156 U/mL) was an independent predictor of early recurrence in the recurrence cohort.

Considering the poor survival observed in the recurrence cohort, especially among patients with early recurrence, high-risk patients may benefit from enhanced multidisciplinary treatment, including neoadjuvant chemotherapy (NAC) and adjuvant chemotherapy, to improve survival after curative resection [28]. Based on the positive results of the Prep-02/JSAP05 study, NAC with gemcitabine plus S-1 is currently considered the standard NAC regimen for rPDAC in Japan [29]. Because the clinical efficacy of NAC with gemcitabine plus S-1 has been demonstrated to improve prognosis and reduce recurrence rates [30,31,32], NAC should be administered to patients with high-risk features identified by our recurrence prediction model. Moreover, more intensive multidisciplinary treatment strategies should be considered for patients predicted to experience early recurrence. Accordingly, preoperative evaluation using a postoperative recurrence prediction model may play an important role in treatment decision-making.

This study had several limitations. First, this was a multicenter, retrospective analysis, which may have introduced a selection bias in treatment decision-making between centers. Second, hospital volume may have influenced the postoperative outcomes. Current guidelines recommend UFS for patients with rPDAC [4]; however, data from the study period are old. Although we developed a recurrence prediction model using a multivariate analysis, other confounding factors may have been present. We performed an internal validation and a decision curve analysis of the prediction model; however, external validation and propensity score matching were not performed, which is an important limitation. Moreover, this study did not sufficiently address the potential risk of model overfitting, which is caused by several factors, such as the presence of noise and a limited size of the training set [33]. Strategies to prevent overfitting may include regularization, cross-validation, and simplifying the model architecture. The applicability of this model should be confirmed through external validation in future studies. Finally, we defined the cutoff value for early recurrence using a *p* value approach as previously reported [4]. However, the current approach did not fully constitute a sensitivity analysis. Therefore, robustness of cutoff justification may be a limitation.

## 5. Conclusions

This study identified prognostic risk factors for recurrence, including CA 19-9 level, tumor size, lymph node metastasis, margin status, and adjuvant chemotherapy, in patients undergoing UFS for rPDAC. We constructed a recurrence prediction model with good discriminative performance based on these risk factors. Moreover, this study revealed that a recurrence-free interval of 5 months was the optimal threshold for early and late recurrence after UFS in patients with rPDAC. An elevated CA 19-9 level was an independent risk factor for early recurrence in the recurrence cohort.

## Figures and Tables

**Table 1:** Clinicopathological characteristics of the 603 patients, stratified by recurrence status.

Variables	Whole Cohort *(n* = 603)	No-Recurrence Cohort (*n* = 222)	Recurrence Cohort (*n* = 381)	*p* Value
*Preoperative factors*				
Gender, men/women				
Men	325 (53.9%)	119 (53.6%)	206 (54.1%)	0.933
Women	278 (46.1%)	103 (46.4%)	175(45.9%)	
Age, years	72 (65–77)	73 (66–77)	71 (65–77)	0.065
BMI, kg/m^2^	21.9 (19.7–24.0)	21.3 (19.4–23.6)	22.1 (19.8–24.3)	0.024
Preoperative biliary drainage	210 (34.8%)	70 (31.5%)	140 (36.7%)	0.215
CA 19-9, U/mL	111 (30–392)	67 (17–266)	149 (47–518)	<0.001
mGPS 0/1/2				
0	418 (69.3%)	150 (67.5%)	268 (70.3%)	0.110
1	137 (22.7%)	59 (26.6%)	78 (20.5%)	
2	48 (8.0%)	13 (5.9%)	35 (9.2%)	
Tumor size, cm	2.3 (1.8–3.0)	2.0 (1.5–2.7)	2.5 (2.0–3.0)	<0.001
Tumor location				
Head	393 (65.2%)	138 (62.2%)	255 (66.9%)	0.250
Body-tail	210 (34.8%)	84 (37.8%)	126 (33.1%)	
Tumor contact with the PV/SMV of ≤180°	174 (28.9%)	48 (21.7%)	126 (33.1%)	0.003
Regional lymph node metastasis	99 (16.4%)	29 (13.1%)	70 (18.4%)	0.110
cStage				
IA&B	53 (8.6%)	26 (11.6%)	26 (6.9%)	0.083
IIA	483 (80.1%)	175 (78.9%)	308 (80.8%)	
IIB	68 (11.3%)	21 (9.5%)	47 (12.3%)	
*Operative factors*				
PD (*n* = 387)	*n* = 387	*n* = 136	*n* = 251	
PV&SMV resection	123 (31.8%)	30 (22.1%)	93 (37.1%)	0.003
Operative time, min	470 (375–560)	484 (371–552)	462 (380–564)	0.794
Estimated blood loss, mL	460 (270–800)	455 (273–793)	465 (270–810)	0.773
DP (*n* = 198)	*n* = 198	*n* = 81	*n* = 117	
PV&SMV resection	3 (1.5%)	1 (1.2%)	2 (1.7%)	1.0000
Operative time, min	272 (219–326)	255 (216–306)	287 (220–352)	0.037
Estimated blood loss, mL	300 (160–572)	270 (145–505)	300 (185–590)	0.318
TP (*n* = 18)	*n* = 18	*n* = 5	*n* = 13	
PV&SMV resection	6 (33.3%)	1 (20.0%)	5 (38.5%)	0.615
Operative time, min	534 (440–625)	415 (326–596)	538 (498–632)	0.085
Estimated blood loss, mL	900 (487–1655)	450 (250–1503)	1100 (670–1690)	0.218
*Pathological factors*				
pT-stage				
T1	48 (8.0%)	33 (14.9%)	15 (3.9%)	<0.001
T2	31 (5.1%)	18 (8.1%)	13 (3.4%)	
T3	524 (86.9%)	171 (77.0%)	353 (92.7%)	
Lymph node metastasis	331 (54.9%)	39 (17.6%)	96 (25.3%)	0.033
R status				
R0	532 (88.2%)	213 (95.9%)	319 (83.7%)	<0.001
R1	71 (11.8%)	9 (4.1%)	62 (16.3%)	
PV/SMV/SpV invasion	135 (22.4%)	39 (17.6%)	96 (25.3%)	0.033
pStage				
IA&B	66 (10.9%)	49 (22.1%)	17 (4.5%)	<0.001
IIA	204 (33.8%)	91 (41.0%)	113 (29.6%)	
IIB	333 (55.2%)	82 (36.9%)	251 (65.9%)	
*Postoperative factors*				
Major complications	135 (22.4%)	51 (23.0%)	84 (22.0%)	0.840
Pancreatic fistula (≥grade B)	113 (18.7%)	41 (18.5%)	72 (18.9%)	0.739
Adjuvant chemotherapy	452 (75.0%)	155 (69.8%)	297 (78.0%)	0.032
S1	409 (90.4%)	152 (98.1%)	257 (86.5%)	<0.001
Gemcitabine	31 (6.9%)	2 (1.3%)	29 (9.8%)	
Others	12 (2.7%)	1 (0.6%)	11 (3.7%)	
Recurrence	381(63.2%)			
Recurrence pattern (*n* = 381)				
Liver	113 (30.0%)			
Peritoneum	43 (11.4%)			
Local	88 (23.4%)			
Lung	45 (12.0%)			
Multiple	83 (22.1%)			
Others	4 (1.1%)			

Values are reported as *n* (%) or median (interquartile range). BMI, body mass index; CA 19-9, carbohydrate antigen 19-9; mGPS, modified Glasgow Prognostic Score; PV/SMV, portal and superior mesenteric veins; SpV, splenic vein; PD, pancreatoduodenectomy; DP, distal pancreatectomy; TP, total pancreatectomy.

**Table 2:** Univariate and multivariate Cox regression analyses of perioperative factors for RFS after surgery in all cohorts (*n* = 603).

Variables	Univariate Analysis		Multivariate Analysis	
	HR (95%CI)	*p* Value	HR (95%CI)	*p* Value
Gender				
Men	0.99 (0.81, 1.20)	0.899		
Female	1.00 (ref)			
Age, years				
≥75	0.94 (0.77, 1.15)	0.567		
<75	1.00 (ref)			
BMI, kg/m^2^				
≥25	1.18 (0.93, 1.51)	0.180		
<25	1.00 (ref)			
Biliary drainage				
Presence	1.31 (1.07, 1.60)	0.008	1.00 (0.81, 1.24)	0.974
Absence	1.00 (ref)		1.00 (ref)	
CA 19-9, U/mL				
≥37	1.86 (1.46, 2.37)	<0.001	1.58 (1.23, 2.04)	<0.001
<37	1.00 (ref)		1.00 (ref)	
mGPS				
2	1.51 (1.07, 2.11)	0.018	1.29 (0.89, 1.82)	0.171
0, 1	1.00 (ref)		1.00 (ref)	
Tumor size, cm				
≥2.2	1.74 (1.42, 2.12)	<0.001	1.59 (1.30, 1.95)	<0.001
<2.2	1.00 (ref)		1.00 (ref)	
Tumor location				
Body-tail	0.85 (0.69, 1.04)	0.113		
Head	1.00 (ref)			
Tumor contact with the PV/SMV of ≤180°				
Presence	1.38 (1.12, 1.70)	0.003	1.14 (0.90, 1.44)	0.259
Absence	1.00 (ref)		1.00 (ref)	
PV&SMV resection				
Presence	1.51 (1.20, 1.88)	<0.001	1.21 (0.94, 1.54)	0.143
Absence	1.00 (ref)		1.00 (ref)	
Lymph node metastasis				
Presence	2.18 (1.78, 2.68)	<0.001	1.86 (1.49, 2.32)	<0.001
Absence	1.00 (ref)		1.00 (ref)	
R status				
R1	1.93 (1.47, 2.52)	<0.001	1.56 (1.18, 2.03)	0.002
R0	1.00 (ref)		1.00 (ref)	
Major complications				
Presence	1.01 (0.80, 1.28)	0.937		
Absence	1.00 (ref)			
Adjuvant chemotherapy				
Absence	1.27 (1.01, 1.59)	0.041	1.54 (1.21, 1.94)	<0.001
Presence	1.00 (ref)		1.00 (ref)	

BMI, body mass index; HR, hazard ratio; CI, confidence interval; CA 19-9, carbohydrate antigen 19-9; mGPS, modified Glasgow Prognostic Score; PV/SMV, portal and superior mesenteric veins.

**Table 3:** Evaluation of cutoff values in determining early and late recurrences based on post-recurrence survival.

Hypothetical Cutoff Value (Month)	Potential Early Recurrence Cohort		Potential Late Recurrence Cohort		*p* Value (Log-Rank)
	*n*	PRS (Median, Months)	*n*	PRS (Median, Months)	
2	14	4.5	367	12.0	6.90 × 10^−4^
3	38	6.0	343	12.0	8.77 × 10^−4^
4	66	5.0	314	13.0	5.64 × 10^−8^
5	95	6.0	286	14.0	1.55 × 10^−8^
6	116	6.0	265	14.0	1.04 × 10^−7^
7	146	7.0	235	15.0	4.34 × 10^−8^
8	165	7.0	216	15.0	1.11 × 10^−6^
9	191	8.0	190	15.0	1.57 × 10^−6^
10	209	8.0	172	15.0	1.32 × 10^−7^
11	223	8.0	158	15.0	1.67 × 10^−7^
12	244	8.0	137	15.0	2.42 × 10^−7^
13	258	8.0	123	16.0	3.79 × 10^−7^
14	269	9.0	112	17.0	5.03 × 10^−7^
15	281	9.0	100	17.0	2.21 × 10^−7^
16	292	9.0	89	18.0	4.86 × 10^−7^

PRS, post-recurrence survival.

**Table 4:** Clinicopathological characteristics of the recurrence cohort (*n* = 381).

Variables	Early Recurrence Cohort (*n* = 95)	Late Recurrence Cohort (*n* = 286)	*p* Value
*Preoperative factors*			
Gender			
Men	158 (55.2%)	48 (50.5%)	0.476
Women	128 (44.8%)	47 (49.5%)	
Age, years	73 (66–78)	71 (64–77)	0.174
BMI, kg/m^2^	22.5 (19.8–24.9)	22.1 (19.9–24.1)	0.400
CA 19-9, U/mL	322 (80–1422)	116 (41–383)	<0.001
mGPS			
0	60 (63.2%)	208 (72.7%)	0.180
1	23 (24.2%)	55 (19.2%)	
2	12 (12.6%)	23 (8.1%)	
Tumor size, cm	2.5 (2.1–3.2)	2.4 (2.0–3.0)	0.015
Tumor location			
Head	67 (70.5%)	188 (65.7%)	0.451
Body-tail	28 (29.5%)	98 (34.3%)	
Tumor contact with the PV/SMV of ≤180°	37 (38.9%)	89 (31.1%)	0.168
Regional lymph node metastasis	24 (25.3%)	46 (16.1%)	0.066
cStage			
IA&B	4 (4.2%)	22 (7.7%)	0.050
IIA	73 (76.8%)	235 (82.2%)	
IIB	18 (19.0%)	29 (10.1%)	
*Operative factors*			
PV&SMV resection	30 (31.6%)	70 (24.5%)	0.180
Operative time, min	428 (309–563)	412 (300–514)	0.256
Estimated blood loss, mL	460 (250–840)	435 (230–733)	0.312
*Pathological factors*			
pT-stage			
T1	1 (1.1%)	14 (4.9%)	0.170
T2	2 (2.1%)	11 (3.8%)	
T3	92 (96.8%)	261 (91.3%)	
Lymph node metastasis	70 (73.7%)	179 (62.6%)	0.062
R status			
R0	82 (86.3%)	237 (82.9%)	0.522
R1	13 (13.7%)	49 (17.1%)	
PV/SMV/SpV invasion	34 (35.8%)	62 (21.8%)	0.009
pStage			
IA&B	2 (2.1%)	15 (5.2%)	0.135
IIA	23 (24.2%)	90 (31.5%)	
IIB	70 (73.7%)	181 (63.3%)	
*Postoperative factors*			
Major complications	25 (26.3%)	59 (20.1%)	0.255
Pancreatic fistula (≥grade B)	19 (20.0%)	53 (18.5%)	0.809
Adjuvant chemotherapy	55 (57.9%)	242 (84.6%)	<0.001

Values are reported as *n* (%) or median (interquartile range). BMI, body mass index; CA 19-9, carbohydrate antigen 19-9; mGPS, modified Glasgow Prognostic Score; PV/SMV, portal and superior mesenteric veins; SpV, splenic vein.

**Table 5:** Univariate and multivariate logistic regression analyses of preoperative factors for early recurrence after surgery in the recurrence cohort (*n* = 381).

Variables	Early Recurrence Cohort (*n* = 95)	Late Recurrence Cohort (*n* = 286)	Univariate Analysis		Multivariate Analysis	
			OR (95%CI)	*p* Value	OR (95%CI)	*p* Value
Gender						
Men	48 (51%)	158 (55%)	1.31 (0.51, 1.31)	0.476		
Female	47 (49%)	128 (45%)	1.00 (ref)			
Age, years						
≥75	39 (41%)	94 (33%)	1.42 (0.88, 2.29)	0.172		
<75	56 (59%)	192 (67%)	1.00 (ref)			
BMI, kg/m^2^						
≥25	23 (24%)	54 (19%)	1.37 (0.78, 2.39)	0.302		
<25	72 (76%)	232 (81%)	1.00 (ref)			
Biliary drainage						
Presence	39 (41%)	101 (35%)	1.27 (0.79, 2.05)	0.328		
Absence	56 (59%)	185 (65%)	1.00 (ref)			
CA 19-9, U/mL						
≥156	67 (71%)	117 (41%)	3.45 (2.09, 5.69)	<0.001	3.28 (1.95, 5.51)	<0.001
<156	28 (29%)	169 (59%)	1.00 (ref)		1.00 (ref)	
mGPS						
2	12 (13%)	23 (8%)	1.65 (0.78, 3.46)	0.217		
0, 1	83 (87%)	263 (92%)	1.00 (ref)			
Tumor size, cm						
≥2.7	39 (41%)	83 (29%)	1.70 (1.05, 2.75)	0.032	1.21 (0.72, 2.02)	0.462
<2.7	56 (59%)	203 (71%)	1.00 (ref)		1.00 (ref)	
Tumor location						
Head	67 (71%)	188 (66%)	1.24 (0.75, 2.06)	0.451		
Body-tail	28 (29%)	98 (34%)	1.00 (ref)			
Tumor contact with the PV/SMV of ≤180°						
Presence	37 (39%)	89 (31%)	1.41 (0.87, 2.28)	0.168		
Absence	58 (61%)	197 (69%)	1.00 (ref)			

BMI, body mass index; CA 19-9, carbohydrate antigen 19-9; CI, confidence interval; mGPS, modified Glasgow Prognostic Score; OR, odds ratio; PV/SMV, portal and superior mesenteric veins.

**Table 6:** A summary of studies reporting nomograms for predicting recurrence after surgery.

Authors	Year	Study Design	Sample Size	Outcomes	Predictive Parameter	AUC/C-Index
Kim et al. [5]	2020	Single-center retrospective, resectable PDAC	Total (*n* = 753) Training (*n* = 631)	Early recurrence (within 12 months)	CEA, CA 19-9, NLR, PLR, tumor size, PV-SMV abutment, differentiation	AUC: 0.655 C-index: 0.665
Li et al. [16]	2021	Multicenter retrospective, resectable PDAC	Total (*n* = 220) Training (*n* = 153) Validation (*n* = 67)	1-year recurrence 2-year recurrence	Radiomics score derived from combined intratumoral and peritumoral CT radiomics features, CA 19-9	AUC: 1-year recurrence 0.916 (training) 0.764 (validation) AUC: 2-year recurrence 0.872 (training) 0.773 (validation)
Xiang et al. [17]	2023	Multicenter retrospective, resectable PDAC	Total (*n* = 435) Development (*n* = 257) Internal validation (*n* = 111) External validation (*n* = 67)	Early recurrence (within 12 months)	Deep learning model output (CT-based), cN stage, arterial involvement	AUC: 0.855 (development) 0.752 (internal validation) 0.741 (external validation)
Man et al. [6]	2024	Single-center retrospective, resected PDAC	Total (*n* = 220) Training (*n* = 604) Validation (*n* = 222)	Early recurrence (within 12 months)	Clinical symptoms, Charlson age–comorbidity index, tumor size, preop CEA, preop CA 19-9	AUC: 1-year recurrence 0.711 (training) 0.730 (validation) C-index: 0.682 (validation)
He et al. [18]	2024	Single-center retrospective, resected PDAC	Total (*n* = 282)	Early recurrence (within 12 months)	Perineural invasion, pTNM stage, preop CA 125, postop CA 19-9, postop CEA	AUC: 0.774
Guan et al. [19]	2025	Multicenter retrospective, resectable PDAC	Total (*n* = 493) Training (*n* = 284) Internal validation (*n* = 123) External validation (*n* = 86)	Early recurrence (within 6 months)	Integrated deep learning radiomics feature score, cN stage, CA 19-9	AUC: 0.941 (test) 0.920 (validation)
Haehira et al. [20]	2025	Single-center retrospective, resected PDAC	Total (*n* = 146)	Early recurrence (within 6 months)	NLR, DUPAN-2, tumor size	AUC: 0.739
Our study	2026	Multicenter retrospective, resectable PDAC	Total (*n* = 603)	Recurrence	CA 19-9, tumor size, pN stage, margin status, adjuvant chemotherapy	AUC: 0.72–0.75

AUC, area under the curve; CA 19-9, carbohydrate antigen 19-9; CEA, carcinoembryonic antigen; CT, computed tomography; DUPAN-2, Duke pancreatic monoclonal antigen type 2; NLR, neutrophil–lymphocyte ratio; PDAC, pancreatic ductal adenocarcinoma; PLR, platelet-to-lymphocyte ratio; PV-SMV, portal vein–superior mesenteric vein.

## Data Availability

The original contributions presented in this study are included in the article. Further inquiries can be directed to the corresponding authors.

## Supplementary Materials

The following supporting information can be downloaded at: https://www.mdpi.com/article/10.3390/cancers18071181/s1, Supplementary Digital Content S1.
